# Reductions in Blood Lead Level Screening During Peak COVID‐19 Restrictions and Beyond

**DOI:** 10.1002/puh2.70021

**Published:** 2025-02-25

**Authors:** Meghan L. McCarthy, Jonah Bradenday, Elizabeth Chen, Mark R. Zonfrillo, Indra Neil Sarkar

**Affiliations:** ^1^ Warren Alpert Medical School of Brown University Providence Rhode Island USA; ^2^ Brown Center for Biomedical Informatics Providence Rhode Island USA; ^3^ Department of Emergency Medicine Warren Alpert Medical School of Brown University Providence Rhode Island USA; ^4^ Department of Pediatrics Warren Alpert Medical School of Brown University Providence Rhode Island USA; ^5^ Rhode Island Quality Institute Providence Rhode Island USA

**Keywords:** health information exchange, lead exposure, lead screening, pediatric primary care, screening, well child care

## Abstract

**Background and Objectives:**

Among the multitude of health effects on children associated with the COVID‐19 pandemic, there have been significant interruptions in the provision of routine pediatric primary care, including blood lead level (BLL) screening. We aimed to investigate trends in BLL screening before and during the pandemic era using patient‐level electronic health record data extracted from CurrentCare, Rhode Island's statewide health information exchange (HIE).

**Methods:**

De‐identified data were analyzed from CurrentCare for the study period January 2018 to December 2021. We utilized ATLAS, a web‐based analytics platform from the Observational Health Data Sciences and Informatics (OHDSI) community, to extract and stratify BLL by variables of interest from the CurrentCare data, standardized to OHDSI's Observational Medical Outcomes Partnership common data model.

**Results:**

A decrease in BLL screening occurred in the spring of 2020, aligning with initial periods of shelter‐in‐place in response to the novel coronavirus outbreak; there was a 48% decrease comparing quarter 2 (April to June) of 2019 and 2020. BLL screening rebounded in the summer of 2020, however, it remained 16% lower overall in 2020 than in 2019. In 2021, BLL screening fell again to 23% lower than in 2019. Although overall numbers of BLL screenings were reduced, the proportion of abnormal BLLs was higher, particularly in the range of 3.5–5.0 µg/dL.

**Conclusions:**

Leveraging statewide HIE data, we found that significant deficiencies in BLL screening remain unresolved since the beginning of the COVID‐19 pandemic. The disruption of children's lives by the COVID‐19 pandemic appears to have greatly affected lead screening and exposure in Rhode Island.

## Graphical abstract

Using a dataset with over 20,000 patients from a statewide health information exchange, we investigated trends of lead screening before and during peak COVID‐19‐related restrictions. We found marked reductions in lead screening in 2020 that persisted into 2021, reflecting concerning trends of decreased utilization of pediatric primary care.

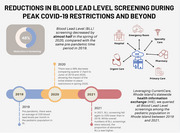

## 1 Background

Childhood lead poisoning is an important environmental health issue, and the efforts to decrease exposure through widespread policies in reducing lead levels in common household items, such as toys, canned foods, and paint, have led to major successes in improving public health. Still, environmental lead exposure remains an important public health issue, necessitating systematic methods for early childhood screening for timely identification and intervention of lead exposure [1]. Although the effect of lead on healthy development is dose‐dependent, even low lead levels are associated with negative and irreversible long‐term effects on cognition, learning, and behavior [2, 3]. In 2021, the Centers for Disease Control and Prevention (CDC) further decreased the standard for a blood lead reference value from 5.0 to 3.5 µg/dL, representative of the fact that there is no “safe” blood lead level (BLL), and that early identification and intervention are critical in reducing the long‐term effects of neurotoxicity associated with lead poisoning [4].

Universal lead screening is state mandated in Rhode Island (RI) with at least two blood lead screening tests on all children by 3 years of age. State‐sponsored guidelines also recommend screening up to age six among children who did not receive both blood tests before age three. Children identified as having elevated lead levels (now defined as ≥3.5 µg/dL) are tracked by the Healthy Homes and Lead Poisoning Prevention Program through the RI Department of Health (RIDOH) to coordinate efforts to reduce lead exposure and prevent further harm from lead exposure [5].

Among the multitude of negative health effects on children associated with COVID‐19 pandemic, an important one was the significant interruption in the provision of routine pediatric primary care. A national report by the CDC found a 34% reduction in the number of BLLs in the period of January 2020–May 2020 as compared to the same period during the previous year [6]. A significant source of lead exposure is in homes built before 1978, and so the significant increase in time children spent in their homes during the COVID‐19 may have further exacerbated the issue, with children spending more time exposed to toxic lead without having the regular primary care visits to detect and intervene on lead exposure [7].

Health information exchanges (HIEs) leverage information technology to facilitate the sharing of health data across hospitals, outpatient practices, pharmacies, and other entities across otherwise fragmented health systems. HIEs were developed as a method of quality‐improvement to streamline communication of health information [8, 9]. Though still limited by information gaps, confidentiality concerns, and other logistical constraints, they have been studied as potential helpful resources for school‐nurses and in emergency room settings in pediatric populations [10, 11, 12, 13]. Additionally, HIEs offer a wealth of information that can be leveraged to investigate important “real‐world” population health questions.

In this study, we used a dataset extracted from RI's HIE to study trends in pediatric lead screening in the COVID‐19 era. We hypothesized that the time periods of peak COVID‐19‐related restrictions would be associated with reduced BLL screening. We also predicted that there would be increased elevated lead levels during the catch‐up periods following the initial COVID‐19 restrictions.

## 2 Methods

### 2.1 Study Design

We used observational data from the statewide HIE in RI to investigate trends in BLL screening between 2019 and 2021.

### 2.2 Setting

RIDOH and the Rhode Island Quality Institute (RIQI) operate RI's HIE “CurrentCare” and is the state‐designated Regional Health Information Organization. CurrentCare contains electronic health data from 48 data‐sharing partners, including electronic health records (EHRs) from all acute care hospitals in RI in addition to data from numerous ambulatory practices, laboratory facilities, imaging centers, and pharmacies across the state. As of 2023, over 536,000 individuals have opted to share their health data with CurrentCare.

The most common source of lead poisoning in RI is through environmental exposure in housing units older than 1978 due to the presence of lead in paint and soil. Up to 80% of housing stock in RI was built before 1978 [14]. The overall prevalence of lead poisoning in RI (defined by the previous standard of 5 µg/dL or greater) was estimated to be 2.9% in 2018 by RIDOH [15].

### 2.3 Data Source

De‐identified (HIPAA expert determination) data from CurrentCare were provided by RIQI for the study period January 2018–December 2021 using the phenotype defined for the National COVID Cohort Collaborative (N3C) [16]. This COVID‐19 pediatric dataset contained lab‐confirmed, suspected, and possible cases of COVID‐19. These cases were demographically matched (on age group, sex, race, and ethnicity) to controls who tested negative or equivocal for COVID‐19, at a ratio of 1:2 (cases to controls). These data were standardized to the Observational Medical Outcomes Partnership (OMOP) common data model.

### 2.4 Variables and Statistical Methods

We utilized ATLAS, a free, publicly available, web‐based analytics platform that was created by the Observational Health Data Sciences and Informatics (OHDSI) community to facilitate descriptive analysis of patient‐level EHR data [17]. First, we created Concept Sets extracting variables of interest using labels within the OMOP standard data format, using Logical Observation Identifiers Names and Codes (LOINC) for laboratory data. This included “Lead [Mass/Volume] in Venous Blood” (concept ID: 46236017) and “Lead [Mass/Volume] in Blood” (concept ID: 3020331); Lead [Mass/Volume] in Capillary Blood (concept ID: 3028406) was not found in this dataset. This Concept Set was then used to identify a cohort of individuals during the specified period (January 2019 through December 2021). Finally, we used the “Characterization” feature in OHDSI ATLAS to stratify the cohort by demographic variables and timepoints of interest. To identify elevated BLLs, we queried all lab findings (with the above LOINC codes) that had results greater than 3.5 and 5.0 µg/dL, which represent the standards of BLL screening during the study period. Once counts were extracted in aggregate, we analyzed temporal trends and demographic associations of BLL screening and rates of abnormal levels using descriptive statistics and graphic visualizations.

## 3 Results

### 3.1 Study Population

Tables 1 and 2 show demographic factors of the study population of the entire pediatric dataset and those with at least one BLL recorded during 2019–2021. Almost all (97.7%) of those in the BLL screening group were in the 0–4‐ or 5–9‐year‐old age group. The proportion of female individuals was almost exactly half for both the overall study population and BLL group. Nearly one‐third (28.7% overall, 30.1% of those in BLL group) identified as Hispanic/Latinx, which is higher than the overall RI population of 17.6% according to Census data [18]. The proportion of individuals in the youngest age group (0–4 years old) remained relatively consistent each year, with 10,881 0–4‐year‐old individuals in the dataset in 2019, 9780 in 2020, and 11,171 in 2021.

**TABLE 1:** Demographics of the entire dataset and the study cohort (defined by having at least 1 blood lead level [BLL] registered in the electronic health records [EHR]‐extracted data).

	Overall dataset	At least 1 BLL (2019–2021)
Age group		
0–4 years	8291 (36.3%)	2874 (57.4%)
5–9 years	8060 (35.3%)	2012 (40.2%)
10–14 years	5727 (25.1%)	104 (2.1%)
15–19 years	779 (3.4%)	13 (0.3%)
Sex		
Male	11,514 (50.4%)	2511 (50.2%)
Female	11,343 (49.6%)	2492 (49.8%)
Ethnicity		
Hispanic/Latinx	6553 (28.7%)	1519 (30.4%)
Not Hispanic/Latinx	14,180 (62.0%)	2712 (54.2%)
Unknown	2124 (9.3%)	772 (15.4%)
**Total**	22,857	5003

**TABLE 2:** Quarterly blood lead levels (BLLs) between 2019 and 2021, and the percentage change between 2019 (representing a pre‐COVID comparison) and 2020 and 2021, respectively.

	2019	2020	2021	% change (2019–2020)	% change (2019–2021)
Q1 (Jan–Mar)	588	629	587	7.0%	−0.17%
Q2 (Apr–Jun)	684	356	564	−48.0%	−17.5%
Q3 (Jul–Sep)	789	742	531	−6.0%	−32.7%
Q4 (Oct–Dec)	686	569	421	−17.1%	−38.6%
**Total**	2747	2296	2103	−16.4%	−23.4%

### 3.2 Lead Screening

**FIGURE 1:**
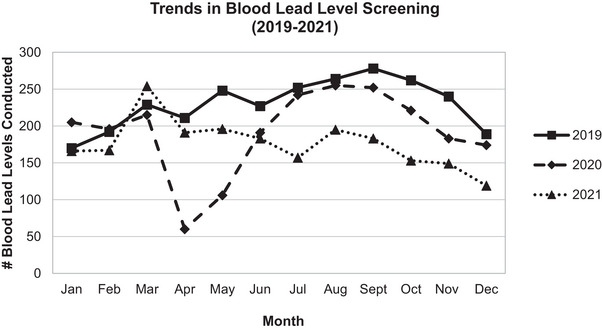
Comparison of monthly counts of blood lead level to demonstrate trends in overall counts during 2019, 2020, and 2021.

Figure 1 compares monthly counts of BLLs in 2019–2021. During 2019, there were an average of 230 BLLs per month. In April and May 2020, there was a precipitous drop in overall BLLs to 60 in April 2020 and 106 in May 2020, representing initial periods of shelter‐in‐place in response to the COVID‐19 pandemic. BLL screening rebounded in the summer of 2020 (6% difference in quarter 2 between 2019 and 2020) and the beginning of 2021 (0.2% difference in quarter 1 between 2019 and 2021). However, BLL was overall decreased in 2020 with a 16% decrease in screening overall in 2021 compared with 2019. BLL screening fell again in 2021, with overall counts of BLL 23% lower compared to those in 2019.

### 3.3 Trends in Elevated BLLs

**FIGURE 2:**
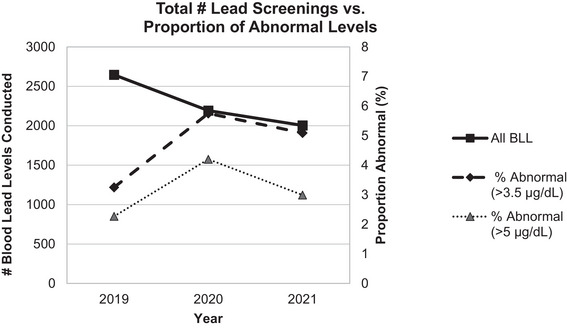
Annual counts of BLL in 2019 through 2021 (primary *y*‐axis) with the proportion of abnormal BLLs (secondary *y*‐axis) during the same period by the two most recent CDC standards (3.5 and 5 µg/dL). BLL, blood lead level; CDC, Centers for Disease Control and Prevention.

Although the total number of BLL screenings decreased over 2019–2021, there was an increased proportion of levels detected above both the current and past CDC standards of 3.5 and 5.0 µg/dL, respectively (Figure 2). Between 2019 and 2021, the rates of elevated BLL by the lower standard of 3.5 µg/dL were 3.25%, 5.75%, and 5.09%, respectively. Comparing 2019–2020 and 2019–2020, the rate ratios of abnormal (using this lower standard of 3.5 µg/dL) to total yearly BLL were 1.76 and 1.55, respectively. In 2019, most abnormal levels (60 out of 68, or 88%) detected fell into the lower standard of less than 3.5 µg/dL. However, the proportion of abnormal levels detected on the higher end of the newer standard (between 3.5 and 5.0 µg/dL) grew each year; by 2021, only 41% (42 out of 102 total abnormal levels) fell into the range between 3.5 and 5.0 µg/dL.

## 4 Discussion

In this study, we compared levels of BLLs in the years 2019–2021 and found that BLL screening was and continues to be dramatically affected by the restrictions put in place due to the COVID‐19 pandemic. Only for 1 month in the study period (March 2021) did BLL counts exceed any month in the pre‐pandemic period of 2019 and January through March 2020. Although there was a slight decrease in the number of individuals in the 0–4 age group between 2019 and 2020, the number of individuals in the 0–4 age group surpassed both years in 2021. This suggests that there are significant deficiencies in lead screening that likely remained overall unresolved. Detection of elevated BLL through lead screening requires timely intervention and follow‐up, which was likely delayed or missed altogether throughout the COVID‐19 pandemic.

In this study, we found that the annual rate of abnormal BLLs increased year‐over‐year between 2019 and 2021. Importantly, the rates identified in this study do not represent incidence, as some abnormal levels may be confirmatory or surveillance tests for the same individual in the database, which were unable to be parsed out due to limitations of the database. However, this is consistent with other research that demonstrated an increased proportion of elevated lead levels during periods of peak COVID‐19 restrictions [19]. When comparing counts of abnormal lead levels according to the previous and current standard for BLL, we found that the proportion of lead levels that resulted in the range of 3.5–5.0 µg/dL increased between 2019 and 2021, suggesting that when abnormal levels were detected in the pandemic period, they were higher on average than pre‐pandemic.

We hypothesize several potential reasons for increasing rates of abnormal lead levels throughout the COVID‐19 pandemic. First, lead poisoning is most commonly due to exposure from deteriorating lead paint in homes built before 1978, when lead paint was banned for residential properties. Up to 80% of housing stock in RI was built before 1978 [14]. Due to school and daycare closures during the early periods of the COVID‐19 pandemic, children had more localized play in and around their homes, potentially leading to higher exposure to lead. It is also possible that those at higher risk are more likely to have been prioritized by their medical providers for BLL screening due to increased risk factors. Nonetheless, the rise in abnormal levels in the context of overall decreased BLL screening is highly concerning and requires further investigation.

As mandated by state law, lead screening in RI most often occurs between the age of 1 and 3 years old [15]. These ages represent an especially important period for routine primary care, namely, due to the provision of vital vaccinations and developmental screenings. Disruptions in lead screening in the COVID‐19 era are one aspect of a broader issue in the disruption of preventive and primary care for pediatric populations. A recent CDC report found that coverage with four vaccines (Measles, Mumps, and Rubella/Polio/Diphtheria, Tetanus, and Pertussis/Varicella) among kindergarten children during the 2021–22 school year remained lower nationally than the 2 prior years, when children entering kindergarten would have received these vaccines prior to the COVID‐19 pandemic. In almost all states, coverage with these four vaccines has declined since 2019 [20]. This is consistent with other research that has demonstrated a drastic drop in vaccination uptake during initial period of the COVID‐19 pandemic, and that these declining rates have persisted [21].

The COVID‐19 pandemic continues to expose and exacerbate significant inequities in pediatric health and healthcare access. Previous studies with geospatial analyses in RI showed that the greatest burden of lead poisoning occurs in neighborhoods with older housing structures and higher levels of poverty [22]. Many of these neighborhoods overlap with neighborhoods that suffered some of the greatest impacts of the COVID‐19 pandemic in RI [23]. For example, the city of Central Falls has the highest rate of childhood poverty in RI (39.4%) with an incidence of first‐time elevated BLL (>5 µg/dL) of 2.7%, compared to the overall state average of 1.7% [14]. Unfortunately, Central Falls was also among the hardest hit cities in RI by COVID‐19, in terms of overall cases and severe disease requiring hospitalization and/or intensive care admission [23, 24]. The impact of COVID‐19 on the provision of routine primary care is yet another way that the pandemic is reinforcing existing health disparities.

### 4.1 Leveraging HIEs to Study Population Health and Medical Screening

An additional aim of this study was to leverage CurrentCare, RI's statewide HIE, to answer an important population health question for a pediatric population. HIEs have many advantages for research and interventions on a population level; they can provide a wealth of information to study population health due to large sample sizes and broad data sources. Recently, Ho et al. utilized CurrentCare's HIE data to study suicidality and mental healthcare utilization among unhoused populations in RI [25]. Ho et al.’s study is one example of how HIEs can be a useful tool to study trends over time for important public health issues that can otherwise be difficult to study, potentially making findings more generalizable than those of a single practice or healthcare entity. Given the relative rarity of many pediatric diseases and distinct ethical challenges of pediatric clinical research, HIEs offer a unique opportunity for pediatric population health research as well.

In addition, HIEs may have important implications in optimizing access to and communication of information from standardized medical screening programs. For example, there have been efforts to utilize HIEs to make the delivery of information from newborn screenings (NBS), a vital tool in detecting and intervening on rare diseases, timelier, and more efficient on a population scale [26, 27]. Aggregating results, such as those of NBS or BLL screening, across healthcare systems could help to make screening programs more robust both for individuals and across populations.

Utilizing health information, however, requires careful attention to data security and de‐identification, which can lead to barriers for its use in research. CurrentCare is currently an opt‐in data sharing system, where patients must provide consent to share their health data, most often during an interaction with the healthcare system (e.g., at an ambulatory visit). This process makes it possible that those represented in CurrentCare may differ from the general population, such as increased representation of those with medical complexity due to more frequent contact with the healthcare system. A legislative change in 2021 will enable the change of CurrentCare's consent model to “opt‐out” (where health data will be shared for all except for those who opt‐out of the system). In addition, due to the rigorous de‐identification of the dataset used for the study reported here, there were some important limitations of our analysis for this study. We were only able to analyze lead levels tested in aggregate, and we were not able to track individual‐level patterns of lead screening nor determine whether individual tests were for screening or confirmatory purposes. Additionally, demographic data pertaining to race, insurance status, exact age (within age group), and geographic data (e.g., ZIP code) were unavailable to be analyzed. Future studies should investigate which populations are at most risk for missing routine lead screening to guide more targeted interventions and follow‐up to resolve deficiencies in lead screening.

## 5 Conclusions

Since early 2020, BLL screening has decreased, whereas the frequency of detected abnormal BLLs appears to have increased among children in RI. The reductions in BLL screening likely represent one outcome of the significant disruptions that continue to impact the provision of pediatric primary care nationally, which have not fully recovered from the initial effects of the pandemic in spring 2020. Leveraging HIE data can provide important insight into statewide trends of pediatric healthcare access and utilization.

## Author Contributions

**Meghan L. McCarthy**: conceptualization, methodology, writing–original draft, writing–review and editing, formal analysis. **Jonah Bradenday**: data curation, supervision, writing–review and editing, software, resources. **Elizabeth Chen**: software, supervision, data curation, writing–review and editing, resources, funding acquisition. **Mark R. Zonfrillo**: conceptualization, methodology, writing–review and editing, supervision. **Indra Neil Sarkar**: conceptualization, methodology, supervision, writing–review and editing, funding acquisition.

## Ethics Statement

The data used were de‐identified (HIPAA Expert Determination) and all analysis of the data occurred in the Stronghold secure computing environment at Brown University. This study is associated with an approved IRB protocol.

## Conflicts of Interest

The authors declare no conflicts of interest.

## Data Availability

This manuscript describes a study involving the use of data from the Rhode Island Department of Health (RIDOH) and Rhode Island Quality Institute (RIQI), which operates Rhode Island's Health Information Exchange and is the State of Rhode Island's designated Regional Health Information Organization. Restrictions apply to the availability of these data, which were used under a data use agreement. Data may be available from the author(s) with the permission of RIDOH and RIQI.
